# Harris' Dictionary

**Published:** 1891-03

**Authors:** 


### Bibliographical.

The first edition of Professor Chapin A. Harris' "Dictionary of Medicine and Dental Surgery" was published in 1849, and a steady and increasing demand for the work encouraged the author to prepare a second edition in 1854, the first having long before been exhausted. The object of the author, at a time when dictionaries of medicine were few in number, and not easily accessible to the dental practitioner and student, was to present a work containing satisfactory definitions and technicalities belonging to Dental Surgery, as well as to the other branches of medicine and to the collateral sciences, in the belief that such a work was greatly needed; and subsequent reflection convinced him that a more extended view of the subject was necessary owing to the fact that the scope of professional education for the dentist had become so widened that general medicine and collateral science had at that time become, to a considerable extent, embraced in the curriculum of dental study. The second edition, therefore, contained, as a result of such reflection, about eight thousand more words than the first edition, and, to prevent an undue increase in the size of the work, the heavier and more elaborate articles were rewritten and abbreviated, and the bibliographical and biographical departments were altogether omitted. All the words, technicalities, and other subjects belonging to Dental Surgery proper, were, however, retained, and all new terms descriptions of subsequent discoveries and improvements in the art and science were carerully added. Numerous synonyms were also introduced, and no important word, in any of the specialities of medicine, was refused a place and a minute and careful definition. The author made, as he stated, free use of the dictionaries, lexicons, and other works on medicine, surgery, pharmacy, physics, chemistry, natural history, etc., which at that time were available. He was also materially assisted by the late Professors A. Snowden Piggot and Washington R. Handy in the preparation of the second edition. The third edition of this Dictionary was not published until 1867, almost seven years after the death of its author, Professor Chapin A. Harris, which occurred in 1860. It was edited by Professor Ferdinand J. S. Gorgas, as have also the succeeding editions, including the present one, who found it necessary to add to this third edition nearly three thousand new words with their definitions, etc. In this edition the doses of the more prominent medicinal agents were added to their definitions, and many obsolete formulæ were omitted, while others were retained for their intrinsic merits, and a number of valuable ones were added. As the work entitled Harris' "Principles and Practice of Dentistry" contained full descriptions of the treatment of diseases of the dental organs, such were briefly referred to, and reference made to the work in which they were to be found. Due acknowledgment was made to the authorities from which interesting matter was obtained in the preparation of the third edition.

Ten years elapsed before another edition of this Dictionary (the fourth) was published, and the object of the editor in preparing the fourth edition was to bring the work thoroughly up to the requirements of the profession of dentistry at that time. Both the dental and medical portions were carefully revised, and many additions made. All new agents of the Materia Medica employed at that time in dental practice were added, so that nearly every one of its seven hundred and forty-three pages contained corrections and additions. The fourth edition was published in 1877, so that a still longer period has elapsed between its appearance and that of the present one.

The object of the editor in preparing this, the fifth, edition of "Harris' Dictionary of Dentistry," has been to make it a more purely dental work than ever before.

Such an object he has endeavored to accomplish by the addition of many hundred words and definitions, which are useful to the dental practitioner and students in qualifying themselves for the study and practice of the science and art of dentistry, and also by the omission of many words and phrases which do not in any manner pertain to dentistry.

The many Medical and other Dictionaries now in use, supply all words and definitions of medicine, chemistry, botany, etc., which are not necessary in a Dictionary of Dentistry. All new words and phrases which have been acknowledged and approved as correct and useful in such a work on dentistry have been carefully selected and used in the present edition, and the obsolete ones, which occupied a large portion of the former editions, have been omitted. The best authorities, such as Gould, Buxton, Quain, Turnbull, Heath, Black, Bloxam, Watts, Fowne, Tomes, and many others, have been consulted, and the object has also been to include in such a selection brief but comprehensive descriptions of the many new operations and appliances which have been generally adopted by the dental profession since the publication of the preceding edition of the work. It is the cherished hope of the editor that the labor and time he has bestowed upon the present edition may be as favorably appreciated as have been his former efforts; and, also, that the new features presented may give this edition of the Dictionary a great superiority over former editions.

---

### Monthly Summary.

